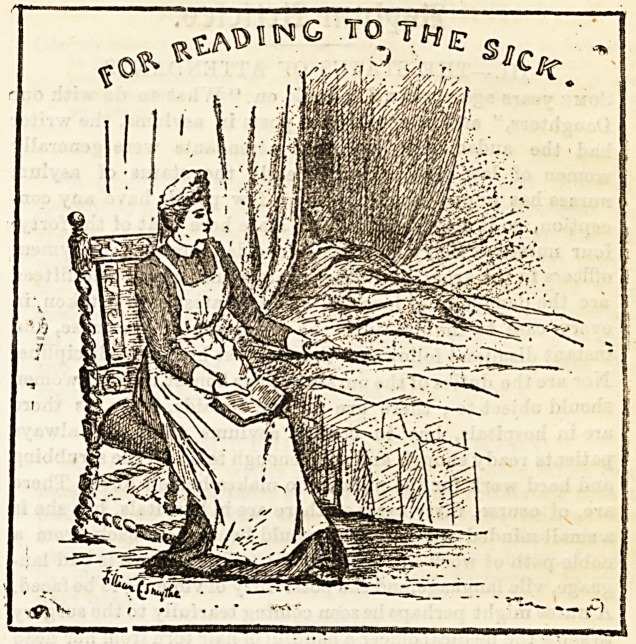# The Hospital Nursing Supplement

**Published:** 1891-07-11

**Authors:** 


### "The Hospital" Nursing Mirror.

Being the Extra Nursing Supplement of "The Hospital" Newspaper.

Contributions for this Supplement should be addressed to the Editor, The Hospital, 140, Strand, London, W.C., and should have the word "Nursing" plainly written in left-hand top corner of the envelope.

### En Passant.

TO MASSEURS AND MASSEUSES.—A number of massage and medical-electrical operators have met to consider the advisability of forming themselves into a society. It was resolved that representatives of the chief massage schools should be invited to attend a larger meeting to consider rules and qualifications for membership.

THE MATRON AT PUTNEY.—Considerable interest has been aroused by the evidence given before the Lords on June 29th with regard to the nursing at the Putney Hospital for Incurables. The following are some of the chief answers given by the Secretary: "What is the position of the Matron? The Matron is the principal officer of the house.—Is she supreme in your absence? She is supreme there altogether in the absence of any of the Committee or of the Secretary; but I do not claim myself to have authority in the house; that is her province. Of course she applies to me for advice, and I take cognizance of everything and anything that goes on.—What is the Matron; is she an English woman? She is a German lady, who has resided in England for the best part of her life.—And has she any knowledge of hospital management? A very good knowledge of it. She was at Sir Patrick Dunn's Hospital in Dublin before she came to us, and she was trained, I believe, at St. Thomas's, and she is a member of the Nightingale Nursing body.—How many trained nurses are there altogether? Five besides the Matron. The second grade are assistant nurses who are not trained. They are to a great extent personal attendants upon the patients under the Matron. I need not say that they have, many of them, acquired a very good notion of nursing and the treatment of invalids, because of being under trained nurses.—Would it not be better to have more trained nurses? They would not be required.—Can you give us any reason why the present Matron, being a foreigner, was appointed to that post? Is there any particular reason why the Committee appointed her? Because of her apparent qualifications and the recommendations that she had.—Were there a great many candidates; was she selected from a great many candidates? No.—Then why did you not invite a great number of applications? This lady was introduced as a person who desired to be a candidate for the place, and the Committee, having seen her and heard all her recommendations, appointed her.—Who was she introduced by? Mainly by a gentleman of the Committee, Mr. George Frederick White, who had known her.—Did you advertise for candidates? I think not at that time.—Is there anything in the nature of a defaulters' book of the nurses kept? No.—Any record of offences committed by the nurses? No. Any offence committed by them would be dealt with as a mistress deals with her servant's faults.—It would be dealt with by the Matron, you mean? By the Matron.—Off-hand? Off-hand it may be.—Perhaps in a passion, if the lady is, as you say, a person of rather infirm temper? Have there been any complaints of the nurses that they had been treated in a high-handed manner by the Matron? Scarcely any.—Have you at this moment a number of cases of bed sores in your hospital? Personally, I only know of one reported as having bed-sores, a person named Lewis.—Are the bed-sore cases mentioned to the Committee every week? The Doctor mentions them in his general report, and says what he thinks is necessary." It is probable that Miss Linacke herself will give evidence in answer to these implied charges.

WORK ABROAD.—Lady Knutsford writes from 75, Eaton Square, to acknowledge the following contributions to the Sierra Leone Cottage Hospital for training native women as nurses: Lady Hay, £5; Mrs. G. W. Lloyd, £3; Marchioness of Salisbury, £10; Hon. Mrs. Cropper, £2; Mrs. Spooner, £3 3s.; J. S. S. Gloucester, £5; Miss James, £2; Hon. Mrs. Way, 5s.; Miss Pocklington, £5; A. C. Worthing, 10s. 6d.; Miss Erle, £10; Nurses' Missionary Association, £10.—Sir Alfred Maloney has presented a minute to the Government pointing out the necessity for training the native women of Lagos as nurses.

THE PRINCESS AND THE NURSES.—The Princess of Wales has done so much for nurses, it is not to be wondered at that the nurses should plead to be allowed to help the fund for Mrs. Grimwood, which the Princess has founded. Here is a special chance for the first and second thousand; let them at least each send a Postal Order for 1s. to the Mrs. Grimwood Fund, care of Miss Knollys, Marlborough House, London, S.W. It is, of course, needless for us to retell how Mrs. Grimwood, after the disaster at Manipur, attended to the wounded at the risk of her own life—one man being shot dead close to her side. Miss Knollys acknowledges the following contributions from nurses, which have given the Princess much pleasure: Nurses M. Thomas, 2s. 6d.; E. Bishop, 2s.; Dugdale, 1s.; no name, 1s.; A. Bryan, 1s.; A. Rays, 1s.; Ella, 1s.; Green, 1s.; Basham, 1s.; E. Evans, 1s.; and A. Spooner, 1s.

SHORT ITEMS.—There are further allegations against Birkenhead Fever Hospital in print, but they are somewhat wild, and have yet to be investigated.—Croydon Guardians are going to expend £35 on giving their nurses a recreation room.—It is proposed to start a district nurse at Chiswick.—Lady Dufferin's portrait has been formally voted to a place in the town hall at Calcutta.—The Vicar of Fulham took the chair at the annual meeting of the Hammersmith and Fulham District Nursing Association, and Mrs. Scharlieb was amongst the speakers.—Cullingworth's Manual of Nursing has been translated into Danish; the whole subject of nursing is very popular in Scandinavia just now.—At the close of the commemoration service of the Order of St. John of Jerusalem, a vote of thanks was passed to the nursing sisters.—The report just issued by the Ipswich Hospital refers with regret to the death on duty of nurse Maria Fiske.—Miss Florence Nightingale has approved the "St. Cecilia Mission" for supplying music to the sick as a method of healing.

THE FIRST AND SECOND THOUSAND.—It is expected that H.R.H. the Princess of Wales will receive the Second Thousand on the morning of Saturday, the 25th inst. The arrangements are not yet definitely fixed, but the nurses of the Second Thousand who desire to be present at Marlborough House should send a post-card intimating the fact to the Secretary of the Royal National Pension Fund, 8, King Street, E.C., on or before Monday next, the 13th inst. It is hoped to arrange for an entertainment for the whole of the nurses who have joined the Royal National Pension Fund on the evening of the 24th inst., at which Lady Cadogan will be asked to preside. Any members of the Fund who desire tickets for the entertainment in question, and those who are resident in the country and will require accommodation in London, should send a post-card to the Secretary stating exactly what are their requirements and intentions. This announcement is made with the object of saving expense, and those nurses who do not communicate with the Secretary may be held to have declined the invitation to the festivities in question.

### Lectures on Surgical Ward Work and Nursing.

By Alexander Miles, M.B. (Edin.), C.M., F.R.C.S.E.

### Lecture XXIX.—BANDAGES FOR LOWER EXTREMITY.

Bandage for the Foot and Leg.—If you examine the shape of this part of the body you shall find that you have first to deal with "a cone" extending from the toes to the heel. This part, then, will require to be covered in by a spiral bandage with reverses. At the heel this cone meets another, that from the heel to the ankle, giving you a "junction of cones," in which case a figure of eight is indicated. At the ankle you have a short "cylinder," for which you employ the simple spiral, and higher up for the cone of the calf you return again to the reversed spiral. Bearing these points in mind, and applying the other rules already given, you stand in front of your patient, having the limb held in the position it is intended to occupy, you carefully apply the wool. Now you have to fix the initial end of the bandage. This is done by making a figure-of-eight turn round the ankle. To do so (*a*) lay the tail of the bandage against the ball of the great toe, and fix it there with your thumb; (*b*) carry the bandage across the dorsum of the foot to the outer malleolus; (*c*) go behind ankle to inner malleolus; (*d*) across dorsum again to the ball of the little toe; and (*e*) across the sole to the point of starting. In this way you fix the bandage, and now you have to begin to cover in the foot. Allow the bandage to go spirally round the foot, leaving one-third of each turn uncovered by the succeeding one, so long as the folds lie evenly. So soon as ever the bandage tends to stray you must begin to make reverses. *To make a reverse* neatly three points are to be attended to: First, to fix the part of the bandage already applied by pressing on it with the thumb of the disengaged hand; second, to free about three inches of the tail, and to allow this to remain *perfectly loose;* then, third, turn the head of the bandage down and allow the loose tail *to fall* into position. Don't try to twist it into position or you will fail to make a neat reverse. Now pull the bandage tight, and proceed as before, repeating the reverses, keeping them all in the same line, and rather towards the outside of the foot, until the heel is reached. Now the figure-of-eight is to be made. Instead of reversing let the bandage go across to the external malleolus, then round the back of the ankle to the internal malleolus, then over the dorsum, keeping the crossing in the same line as the previously made reverses, and passing round the outer border of the foot, travel under the sole to the point at which you started your figure-of-eight. This is to be repeated until the heel is sufficiently covered in, and then the ankle and calf are to be bandaged after the appropriate methods. To finish the bandage a figure-of-eight turn is made round the upper part of the calf, and the terminal end fixed by means of a safety pin inserted parallel to the edges of the bandage, or by tearing the end into two tails, and tying these in a reef knot.

Of course, should it be necessary to cover in *the whole of the lower extremity* right up to the groin, the bandage just described, instead of being finished at the upper end of the calf, is continued upwards over the knee, which is covered in by a series of figure-of-eight loops, on to the thigh, where the reversed spiral is employed; and to finish and secure the terminal end, a figure-of-eight turn is made round the waist, or, more correctly round the *pelvis*.

To Cover in the Heel Alone.—This is best done by what is called a "divergent spica," that being merely a modification of the figure-of-eight. It is called divergent, because the first turn covers in the most prominent part of the heel, and from it the succeeding turns diverge. As elsewhere, you must first fix the bandage, and to do so place the tail over the external malleolus, carry the bandage downwards across the sole to the internal malleolus, thence across the dorsum and round the ankle, catching in the tail with which you started. The bandage is now fixed, and the head is over the inner ankle. Carry it straight across the tip of the heel, and in doing so you will leave small pockets above and below. The next turn goes a little lower than the last, catching up and covering the corresponding pocket, and the succeeding one going higher similarly disposes of the upper pocket. With one or two more turns diverging from the tip of the heel that part can be effectually covered in. The heel may also be covered in by a loop bandage.

Bandages for the Knee.—(*a*) *Divergent Spica.*—This is employed when it is desirable to permit of a slight amount of movement at the knee joint, as the different layers of bandage glide over one another like the plates of scale-armour. Slightly flex the limb, and begin by making a turn round the most prominent part of the knee, a second turn over-lapping the lower part of that, and a third overlapping the upper part. Succeeding turns continue to diverge till the whole joint is covered in. It is obvious that the anterior aspect of the knee is the less firmly supported by this bandage, but the presence of the patella renders many layers unnecessary there.

(*b*) *Convergent Spica* is simply a figure-of-eight put on, so that the successive turns converge towards the centre of the patella. It is used when fixature of the joint is aimed at, or to approximate the fragments in fracture of the patella.

(*To be continued.*)

### Notices.

The Mayor of Birmingham invites the nurses and managers of the Birmingham Midland Counties hospitals and kindred charities to a meeting at the Council House at 3 p.m. to-day (Saturday), when the founder of the Royal National Pension Fund for Nurses will give an address and be prepared to answer any questions as to the advantages of the Fund to nurses and officials who invest their savings in it. Having regard to the Princess of Wales's visit to Birmingham next week, and the great personal interest she takes in the Fund, this meeting is well timed and should prove helpful to the nurses and managers of the many institutions in the Midlands.

The Superintendents of the Blackheath and Richmond Nursing Institutions give a certificate to those nurses who complete three years of satisfactory work on their private nursing staff, and at the completion of five years a silver badge.

### Prize Invalid Jackets.

WE give illustrations this week of the two bed jackets which took prizes last Christmas. The first one is made of undyed wool bound with narrow red ribbon. This saves any ridges such as hems make, and which are uncomfortable to lean on. The sleeves unbutton all the way down, and the shoulders unbutton, so that it would be perfectly easy to put on a poultice, or to massage the patient, without removing the jacket as a whole. There is a nice big pocket.

The jacket which took the second prize is made of dark red flannel. It unbuttons down each side and on the shoulders. The sleeves are gathered at the wrist by a piece of elastic to prevent draughts, and there is a piece of ribbon to tie the jacket in at the waist. It measures twenty-four inches from neck to hem, fifty-four inches round the bottom, eighteen inches round the neck; the sleeves are twenty inches long. Both in size and style it is a woman's jacket, whereas No. 1 was undoubtedly meant for a man. We are sending both jackets to Sister Frances, of St. Luke's Home, Vancouver, in answer to her appeal which we printed last week.

### Presentation.

MISS WALMSLEY, Lady Superintendent, retired from the Home for Incurables, Liverpool, on Thursday, July 2nd, where she has worked for nearly sixteen years. She received, in recognition of her valuable services, an address from the Ladies' Committee, with a large case containing silver teapot, sugar basin, and cream jug; from her loving and grateful patients, handsome silver travelling flask; from the nurses, large photo of themselves, in a frame; besides other valuable presents from numerous friends.

### FEAR.

THERE are two kinds of fear in life; one a slavish dread of everything out of the common, whether it be in fiction, a prancing horse, or the ridicule of our companions. The other is a safeguard. We do not expose ourselves to unnecessary danger lest we alarm or hurt those who love us; we keep in many a sharp word or rough action from the same cause, and in holy things it prevents our "rushing in where angels fear to tread."

We must strive against the former, which is occasioned by half-imaginary troubles, and try to face them boldly that they may not get the better of us, and make us very selfish.

Many persons will stand out against the urgent wishes of doctor and friends; they will *not* go to a hospital to have the best advice and skill in treatment because they are afraid of being among strangers, or of having "experiments" tried on them, as they term it, and so they wear out relations and friends with fatigue and anxiety and expense from want of a little courage and self-control. Who has not seen the wry face and decided refusal to take an unpleasant draught, or a sufferer go through a martyrdom rather than have a tooth drawn? Cowardice in both great and little things interferes with our own and other people's comfort and success in business. We hesitate to take an important step in life, the opportunity is gone when we have got our heart up to the occasion; we lose a train because we cannot persuade ourselves to cross a busy thoroughfare. We dread heat and cold, tempests and driven cattle, till our lives are a burthen to us. The remedy for all these real and fancied trials is faith in a God who promises to watch over us always. A sparrow does not fall to the ground without his knowledge; how much better are we and dearer to Him than many sparrows?

What care a loving mother takes of her little one. Our loving Father is wiser, greater, more powerful than any human parent, and will protect and guide us in all troubles and dangers if we trust in Him. Let us cast away all fear and rest in His love He will give us all the strength and courage we need. How happy shall we be if we strive to love Him perfectly, "for perfect love casteth out fear because fear hath torments.

"Fear not, oh! trembler, God is strong to save
From pain and grief and every woe of mortal;
He soothes our sorrows, and with love doth pave
Our earthly road, to lead us to heav'n's portal."

### Asylum Articles.

### III.—THE STATUS OF ATTENDANTS.

Some years ago appeared a book on "What to do with our Daughters," and in speaking of posts in asylums, the writer had the audacity to say that attendants were generally women of low class. How greatly the status of asylum nurses has improved of late years few people have any conception, so it may be as well to state here that of the forty-four nurses at Berrywood, there are daughters of clergymen, officers in the army, and other professional men; and fifteen are the daughters of farmers. The greatest care is taken in every case to inquire into the character of the nurse, and instant dismissal follows on the slightest breach of discipline. Nor are the duties of the nurses such as honest, upright women should object to; there are no ward-maids, truly, as there are in hospitals, but in county asylums there are always patients ready enough and sane enough to do all the scrubbing and hard work for the nurse who makes herself liked. There are, of course, dirty cases as there are in hospitals, but she is a small-minded woman who would be turned back from a noble path of work by such details. Then there is bad language, vile language, and the possibility of violence to be faced. A nurse might perhaps be seen coming tearfully to the surgery to show the medical officer a handful of hair torn from her head by a patient. Nor is it a pleasant sight, these rooms full of women whose faces are marred by a wild or vacant look. All these are objections, and many more could be urged, but it is not worth while in face of the fact that Berrywood never has to advertise for a nurse; there is always a list of suitable candidates waiting for the next vacancy. For you see, if there are *pros*, there are also *cons.*; to begin with, asylums are usually in the country, where fresh air can be enjoyed; the chances of promotion are excellent; and the life is more varied than that of a hospital nurse. The attendant goes out walking with her patients every afternoon, she takes part in the weekly concert, she acts in the theatricals, and goes into fits of laughter when the medical officer plays Mrs. Jarley. The life is home-like, the duties varied, and the patients become attached to the nurse, and the nurse to the patients. As for the solid satisfaction of knowing that the work among these stricken and afflicted ones is worth the doing, that can cheer the nurse's heart and write a perpetual smile on her face. When Christ was on earth He gave thought to the sick, but also to the demoniacs, the epileptics, and the lunatics. All three classes are mentioned as having been healed by Christ when they were brought to Him; but nowadays we are apt to reserve all our sympathy for those diseased in body, and to hide away and forget those that are diseased in mind. Did Royalty ever lay the stone or open the building devoted to the mentally afflicted? In all the thousand-and-one benefits now conferred on hospital nurses there are but few in which asylum nurses can participate; certainly they have their part in the Pension Fund, and when the Second Thousand are received at Marlborough House the most striking group present will be the ten members from Berrywood in their military-looking uniform.

When the present craze for hospital nursing subsides, perhaps more ladies may be brought to see that equal honour and equal labour can be had in asylums. Indeed, it is greater honour, for those now entering are the pioneers as it were of a better state of things. All talents are brought into play in an asylum: a trained voice, a knowledge of music, histrionic art, are all appreciated; but most of all is desired women who combine mental strength with gentle manners, who have tact and are cheerful in their speech and ways. The noble woman and the perfect lady have a fine field before them in introducing into asylums all the refinement, thought, and tender skill which have so immeasurably brightened our hospitals.

### American News.

Dear Mr. Editor,—You say your correspondents are always asking for particulars of nursing here in New York. Well, they have only to judge by things in England; there is no great difference between the new and old world in nursing matters. We have rather higher pay here, but living is dearer, and we work through directories and take our own earnings instead of institutions. In *Harper's Bazaar* this week there is a good article on "Trained Nurses." It says: "After six months, the senior assistant becomes a head nurse, and has the responsibility of a ward and of two assistants. She is now doing the work most valuable to the hospital, and her pay is raised to sixteen dollars a month. She continues to receive instruction in the school, and also is called upon to give instruction to her assistants and to the beginners. In this capacity she remains a year, and at the end of that time, if she pass a satisfactory examination in the presence of the Executive Committee of the hospital, she is given a diploma as a trained nurse under the seal of the hospital. She is now qualified to practise her profession among private patients. At the New York Hospital, and, indeed, at all of the hospitals which have training schools, a register of graduates is kept, and anyone wishing to employ a nurse can go to any of these institutions and receive the addresses of nurses not at the moment engaged. If a trained nurse were engaged all the year round, she would be able to make very good wages, as she gets, when she is employed, from fifteen to fifty dollars a week, the average being about twenty-five. And during the time of employment she has no board to pay, though, of course, her rent goes on all the same. A good nurse—one in whom several doctors, for instance, have confidence—can be pretty sure of making six hundred dollars a-year."

When Dr. Josepha Williams was in England she called on you, I know, and I guess that, in common with most folk, you admired her very much. She is so young and elegant—one scarcely knows how to call her "doctor." Well, she and another lady doctor have started a Sanatorium at 1,542, Pearl Street, Denver, a fine residence, in a fine situation. They have got trained nurses on hand, and their inclusive charge is from twenty-five to sixty dollars a-week.

A nurse of the war of 1812, Mrs. Elizabeth Sands, died at Baltimore lately, aged 101 years. She was a person of great activity, and it is said that after having passed her one-hundredth birthday she would go up and downstairs thirty times a day.

The *Nightingale* lately gave an interesting answer to an English nurse who asked about emigrating to New York. It said that "You should have enough money to allow of some waiting for cases, at least at first. It is a mistake to turn to other work during these intervals. We have known of nurses who have become housekeepers or companions in order to help themselves along before getting established. This is always a detriment to subsequent prosperity. That which would be admirable and praiseworthy in other departments of labour is here a reproach. The nurse cannot turn her hand to unprofessional work when out of employment without a loss of public esteem. To any woman wanting to establish herself here in nursing we would say do not attempt it unless you can bring with you the equivalent of 200 dols., or at least 100 dols. in ready money. With this proviso you have a fairly good chance of success. The profession is not yet overcrowded here, although we have times of peculiar immunity from sickness when many of our best nurses even are unemployed."

The first meeting of the Brooklyn Association of General Hospital Graduate Nurses took place Monday, April 20th, at the Johnston Building, Flatbush Avenue. This meeting was for the purpose of organising a Society of Trained Nurses, to meet once every month, in order to listen to a good lecture, and to have an opportunity for bringing up and discussing any matter concerning the profession. We are a good deal more given to small societies and clubs than they are in England; in fact, we are more sociable and less stiff.

### The Sarah Acland Home for Nurses, Oxford.

In our issue of April 25th we wrote concerning the arrangements for retiring allowances in regard to the nurses of the above home: "We were sorry to hear that the Committee had decided not to formulate any definite system of pensions, but to give each nurse a sum per annum to do what she liked with. We venture to think that this plan is neither desirable nor fair. Not desirable, because it fails to bring to bear, in the interests of the nurses, the experience and knowledge of the Committee so as to secure an adequate provision for each nurse against sickness or old age. Not fair, because nurses are often drawn from a class which makes the members of their families regard them as in a position of relative prosperity, and so causes the friends to deplete a nurse's earnings by constant drains upon her small resources. Hence it is quite possible that, in spite of an institution giving each nurse on its staff a sum, say, of £4 a-year, for the purposes of a pension if it be left absolutely at the nurse's own disposal, it will be found that in the day of sickness and old age not 5 per cent. of the nurses belonging to such an institution will have made any adequate provision, or have funds in hand to pay for their maintenance. The Managers of the Acland Home take so keen an interest in its welfare and its staff, that we hope it is not too late for them to reconsider this proposal, which we are confident will be found in practice to fail in its purpose, and so to prove unsatisfactory to the institution as well as to the nurses employed." With reference to this statement, the Secretary, Mr. E. S. Turner, writes, on behalf of the Committee, requesting us to publish the words of the following resolution passed by the Committee of the Institution on February 11th last: "It was agreed that from January 1st, 1891, £2 per annum should be allowed to each nurse after five years or more in the Home who can show that she is already making provision by insurance towards her support when she shall be incapacitated for the work of a nurse; such sum of £2 to be payable in equal half-yearly portions, and the first payment to be made six months after the completion of the five years or more of service." It seems to us that this resolution supports the objections we originally urged.

### Appointments.

[It is requested that successful candidates will send a copy of their applications and testimonials, with date of election, to THE EDITOR, The Lodge, Porchester Square, W.]

MIDDLESBROUGH FEVER HOSPITAL.—Miss Esther Jones, Matron of the Borough Hospital, Devizes, Wilts, has been elected Matron of the Fever Hospital, Middlesbrough. Miss Jones was trained at the Royal Southern Hospital, Liverpool, and has since worked at the Cardiff Infirmary and Monsall Fever Hospital.

MARSDEN HOSPITAL. — Nurse Janet Macdonald, of the Huddersfield Nurses' Home, has been appointed Matron of the Cottage Hospital at Marsden, near Huddersfield. She completed a year's training at Bootle Borough Hospital in November last, after which she took two months' training at Queen Charlotte's Hospital. Since February she has been on the staff of the above Home.

SURREY CONVALESCENT HOME.—Miss R. Napper has been appointed Matron of this Home for fifty male patients at Seaford; it will be opened on July 23rd. It is only a few weeks since we noted Miss Napper's appointment to the Santa Claus Home, and there must surely be something wrong with the management of the latter institution, since such an excellent officer resigns so rapidly.

### Everybody's Opinion.

[*Correspondence on all subjects is invited, but we cannot in any way be responsible for the opinions expressed by our correspondents. No communications can be entertained if the name and address of the correspondent is not given, or unless one side of the paper only be written on.*]

### THE GARDEN PARTY.

"A QUEEN CHARLOTTE'S NURSE" suggests that those of her fellow nurses, who might find it more convenient to appear at Marlborough House in outdoor costume, should wear over their uniform a cloak and bonnet of light grey; the cloak, Russian shape, which can be obtained at a very moderate price from Messrs. Denton and Holbrook, Gloucester; the bonnet, plain straw trimmed with silk to match and white strings. The above costume meets with the approval of their estimable Matron, Mrs. Phillips.

### DEWSBURY GENERAL INFIRMARY.

THE SECRETARY writes: My attention has been called to a paragraph in your issue of last Saturday, intimating that a patient had died from the effects of carbolic acid at the Dewsbury Infirmary. This was not the case. The unfortunate misadventure happened at the Union Infirmary. I shall be pleased if you will kindly make the correction in your next issue.

### THE BRASSEY HOME.

"BEATRICE" writes: I shall be glad if you will allow me a little space in THE HOSPITAL to express my opinion of the Brassey Holiday Home, St. Leonards-on-Sea. I consider it a boon to everyone, but especially to nurses. They have every consideration shown them. The rules are not too strict. I spent a pleasant holiday, and shall be very pleased to go again.

### Wants and Workers.

[Under this heading, we propose to try whether we can be useful to our readers in making the wants of some known to others who are willing to do what work they can to aid the great cause of curing and cheering the sick. Wants can only be inserted from those who are connected with some institution or association, or who are willing to have their full name and address printed.]

*Sister Frances* acknowledges, with thanks, bed-jackets, books, charts, and "letter with very generous response," in answer to her appeal for help for St. Luke's Home, Vancouver.

"*The Hospital.*"—Weekly copies from August, 1890, to March, 1891, will be sent free to any institution or nurse wanting them.—*K.M.*

*Pictures Wanted.*—The Lady Superintendent of King's Lynn Hospital, Norfolk, would be glad of pictures, scrap-books, and red jackets for her wards. Will anyone willing to help please write to the Lady Superintendent?

*Nurse Winter* sends thanks to "An Ex-Nurse" and Miss Graham for cards received.

### Notes and Queries.

TO CORRESPONDENTS.—1. Questions or answers may be written on post-cards. 2. Advertisements in disguise are inadmissible. 3. In answering a query please quote the number. 4. A private answer can only be sent in urgent cases, and then a stamped addressed envelope must be enclosed. 5. Every communication must be accompanied by the writer's full name and address, not necessarily for publication. 6. Correspondents are requested to help their fellow nurses by answering such queries as they can.

**Queries.**

(19) A Country Rector's Wife will be glad to know the price of the "Zona Elastic Belt"; how it is fastened, and if made in different sizes. She will be much obliged for any information respecting the "Belt."

**Answers.**

(18) You can get a district nurse's basket from Messrs. Southall, of Birmingham.

*A. B. N.*—If you read the letter we have printed instead of yours, you will see how far more polite it is.

*Hopeful.*—Some day; we are too busy just now.

*C. W. S.*—Miss Meyrick kindly sends word that her present address is care of C. Hooper, Esq., Aylesbury, Bucks; any letters sent there will always find her.

*M. B.*—See THE HOSPITAL for April 4, in which various correspondents said, some that water beds could be mended at any rubber shop, others that nowhere could such beds be mended.

*W. T. M.*—Thanks for your letter; yes, we are always glad of "items."

*Nurse C.*—"Cullingworth's Manual for Monthly Nurses," published by Churchill, price 1s. 6d. Lewis's "Theory and Practice of Nursing" (THE HOSPITAL Office) is just coming out in a new edition, with illustrations; you had better wait for that.

### In a Devonshire Village.

"Porte after stormie seas."—*Spenser*.

It is now many years ago since I was suddenly sent for to nurse a case in a small village in North Devon.

I had just returned from nursing a heavy case in London, and felt at first somewhat aggrieved at being sent off again, without even time to unpack my box, instead of enjoying the few days' rest and quiet to which I own I had been looking forward somewhat eagerly.

But I became quite contented again at the sight of my destination; as you may imagine when I tell you that I, who had never been in Devonshire in my life, now found myself in a most comfortable farmhouse, not far from the village of Lower Southtripton, one of the most picturesque spots in the most lovely county in England.

After the heat and noise of London, the sounds and sights of the sweet country life were a double refreshment to me, and I felt thankful that the lot had fallen to me—for the present at least—in "pleasant places."

My patient needed all my attention. I found him prostrate with continued fever, which had brought him down to that most helpless and piteous condition where all "desire fails," and the feeble strength seems unable to take up the burden of life again. He thanked me for each small service with a grateful look, but seldom spoke. He seemed to be a man who, though apparently only middle aged, had outlived the pleasures and interests of life, and who, now brought low, had no tie to draw him back to life, or to rouse him to a wish to live.

I asked Mrs. Gray, the farmer's wife, for information about my patient; but she could give me very little.

Mr. Newman had come to their house three weeks ago, and had taken a room for two nights. On the day he was to leave, he had been taken ill, and had not since left his room. He had become gradually worse, until the doctor had insisted that a nurse should be sent for. The patient had given no trouble, and had not asked that anyone should be sent for. That was all she could tell me. He appeared to be well off, and his few belongings proved him to be a man of refined and cultivated tastes.

By his bedside I found a small collection of books, with which the sick man had tried to pass the long hours at the beginning of his illness. There were one or two classics in the original, a Greek testament, and a much thumbed book on botany. In one corner of the room lay a bunch of withered flowers, probably some botanical specimens gathered during his last walk.

Some weeks passed before I could see any marked improvement in his state. His weakness continued, but he talked more to me, and seemed pleased to hear me read aloud to him. He also took an interest in the wild flowers which I brought back from my short daily walks, and arranged by on the table by his bedside, and he corrected my pronunciation of the long Latin names, which I found out of his book on botany.

One evening I sat by the open window watching the lengthening shadows fall across the peaceful meadows, and feasting my eyes, which were never tired of gazing, on the fair English landscape which stretched away from the farm to the grey hills, whose tops were glowing in the rays of the setting sun.

I had been reading aloud from Miss Proctor's poems, "A Legend of Provence," and my patient had, as I thought, at last fallen asleep. The room was perfectly quiet, except for the subdued sounds of farmyard life which came up to us at intervals from below.

"We always may be what we might have been," Mr. Newman was repeating to himself a line from the close of the poem I had read to him. I went to his bedside and sat down there.

He turned his eyes towards me.

"I wish that was true, nurse," he said feebly, with a faint smile.

"Is it not possible sometimes?" I asked, pleased that he had followed, and taken an interest in what I had been reading.

"Sometimes, perhaps," he assented, "but not often. We are what we are, what our past history has made us, and who can undo that?"

"I am, as you see nurse, a very lonely old man,"—again that pathetic smile—"but it might have been very different; it might have been."

He repeated the words slowly, as if his memory was busy with the past. There was silence again for a while; then he continued, speaking with an effort:

"I had a son once, who might have been with me now, but I drove him from me with my ungovernable temper and harsh treatment, and now I do not know whether he is alive or dead. I might have been a good father to him, but how can I be to him what I might have been, when I cannot even hope that I shall see him again."

I hardly knew what to say to him. To me it seemed a thing almost incredible that anyone so uncomplaining as he invariably was, so patient in bearing pain and weakness, so considerate for others; so grateful for every service rendered, could ever have been the bad-tempered, violent man he described.

He had evidently suffered much. The deep lines on his face spoke of hard struggles with the enemy, but the calm and peaceful expression he now wore seemed to me to speak of the victory of one who had "fought a good fight."

He was in a mood to talk that evening, and presently went on, more to himself than to me:

"There are some troubles sent from God, and they are easy to bear, because comfort is sent with them. There are troubles from our fellow men, which are also bearable when they arise from no fault of ours, but there are troubles which we make ourselves, and how can we expect to be saved from them?"

I suggested that if his son knew of his wish, that he would come to him at once.

"He was a good boy," he answered, "until I thwarted him in the wish of his life, and then drove him from me. Where he is now I cannot tell. He might come, but I have no right to expect it. I have long ago seen my faults, and I trust have been forgiven, but I cannot escape from the punishment of them. It is just."

The effort to say so much had exhausted him, and the consequence was a wakeful night. There was no word of complaint, but constant tossings and weary restlessness, so painful to witness, and so difficult to soothe. In the morning he was decidedly worse, and the doctor asked me whether he had expressed a wish to see any of his friends.

"He has no one but a son," I said, "and he does not know where he is."

(*To be continued.*)

### Amusements and Relaxation.

**SPECIAL NOTICE TO CORRESPONDENTS.**

**Third Quarterly Word Competition commenced July 4th, 1891, ends September 26th, 1891.**

Competitors can enter for *all* quarterly competitions, but no competitor can take more than *one first prize* or *two* prizes of any kind during the year.

The word for dissection for this, the SECOND week of the quarter, being

"ARIBERT."

| Names. | July 2nd. | Totals. | Names. | July 2nd. | Totals. |
|---|---|---|---|---|---|
| Christie | 117 | 524 | Ivanhoe | 114 | 453 |
| Patience | — | 214 | Weta | — | 147 |
| Agamemnon | — | 367 | Lady Betty | — | — |
| Hope | 130 | 544 | Mortal | — | 76 |
| Reldas | 130 | 540 | Little Eliza | — | 147 |
| Lightowlers | — | — | Dove | — | 95 |
| Nurse J. S. | 117 | 473 | Ladybird | — | 141 |
| Qu'appelle | — | 170 | Psyche | 118 | 476 |
| Jenny Wren | 87 | 434 | Ugug | — | 229 |
| Wyameris | 131 | 540 | Harrie | — | 69 |
| Paignton | 121 | 467 | Grannie | 91 | 427 |
| Theta | — | — | Eale | — | 169 |
| Success | — | 17 | Grimalkin | — | 53 |
| Tired | — | 136 | Nurse G. P. | 89 | 214 |
| M. G. | — | 188 | | | |

**Results of Third Quarterly Word Competition.**

*First Prize*, 15s., is awarded to Hope (Miss Mason, 112, Mildmay Road, N.).

*Second Prize*, 10s., is divided between Reldas (Miss Sadler, 32, Norland Square), and Wyameris (Miss Whaley, Barnsley, Yorks).

*Third Prize*, 5s., is awarded to Christie (Mirs Alice Meadows, Royal Hospital, Putney, S.W.).

N.B.—Each paper must be signed by the author with his or her real name and address. A *nom de plume* may be added if the writer does not desire to be referred to by us by his real name. In the case of all prize-winners, however, the real name and address will be published.

All letters referring to this page which do not arrive at **140, Strand, London, W.C.**, by the *first post on Thursdays*, and are not addressed PRIZE EDITOR, will in future be disqualified and disregarded.

## Figures and Tables

**Figure f1:**
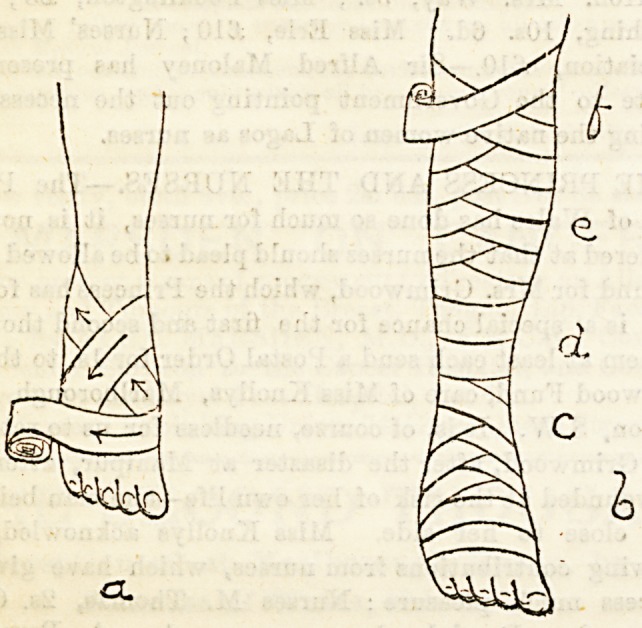


**Figure f2:**
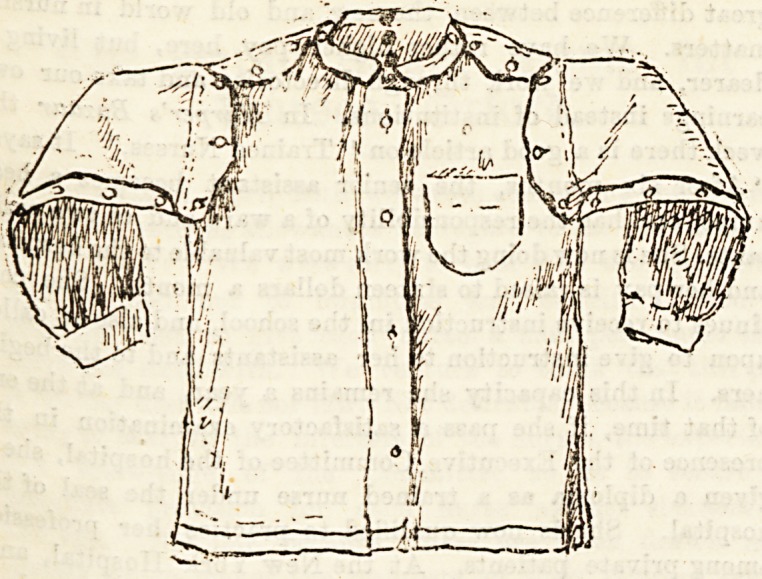


**Figure f3:**
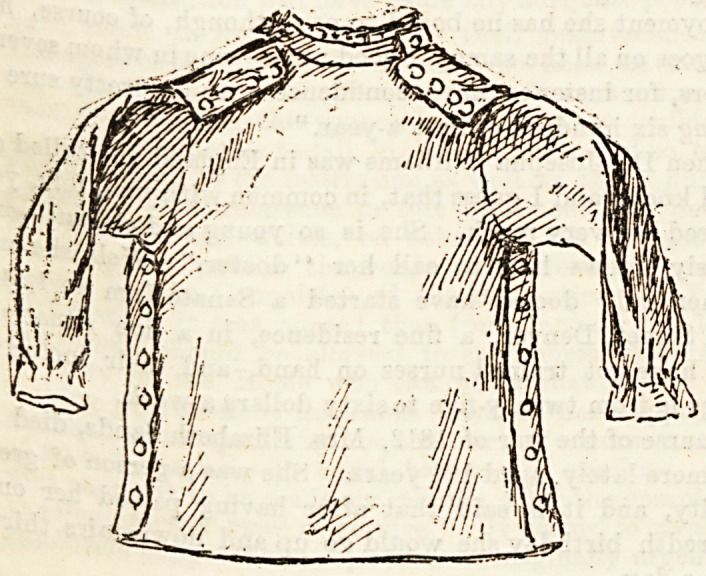


**Figure f4:**